# Efficacy of intratympanic or postauricular subperiosteal corticosteroid injection combined with systemic corticosteroid in the treatment of sudden sensorineural hearing loss: A prospective randomized study

**DOI:** 10.3389/fneur.2023.1138354

**Published:** 2023-04-06

**Authors:** Wen Xie, Niki Karpeta, Jiali Liu, Haisen Peng, Chunhua Li, Zhiling Zhang, Yuehui Liu, Maoli Duan

**Affiliations:** ^1^Department of Otolaryngology Head and Neck Surgery, The Second Affiliated Hospital of Nanchang University, Nanchang, China; ^2^Division of Ear, Nose and Throat Section, Department of Clinical Science, Intervention and Technology, Karolinska University Hospital, Karolinska Institutet, Stockholm, Sweden; ^3^Department of Otolaryngology Head and Neck & Audiology and Neurotology, Karolinska University Hospital, Karolinska Institutet, Stockholm, Sweden

**Keywords:** sudden sensorineural hearing loss, intratympanic injection, postauricular injection, corticosteroid, treatment

## Abstract

**Objectives:**

This study aimed to investigate the efficacy and safety of intratympanic or postauricular subperiosteal glucocorticoid injection combined with systemic glucocorticoid in the treatment of sudden sensorineural hearing loss (SSNHL).

**Methods:**

This study is a prospective randomized controlled study. This study included unilateral SSNHL patients who were hospitalized in our department between January 2020 and June 2021. Patients were randomly divided into three groups (groups A, B, and C). Patients in group A were treated with an intratympanic corticosteroid injection combined with systemic corticosteroid treatment, and patients in group B received a postauricular corticosteroid injection combined with systemic corticosteroid treatment. Patients in group C (control group) were treated with systemic corticosteroid alone. The case number of groups A, B, and C was 311, 375, and 369, respectively.

**Results:**

There was no significant difference in gender distribution, the proportion of left and right affected ears, and the average interval from onset to treatment among the three groups (*P* > 0.05). However, there were significant differences in their average age, distribution of audiogram type, and hearing loss levels among them (*P* < 0.01). Our study shows that there was no significant difference in average hearing threshold improvement before and after treatment in the three groups (*P* > 0.05). Regarding the complications, in group A, 33 patients (10.6%) had a transient vertigo attack during tympanic injection, which lasted for ~1–3 min. In group B, 20 patients (6.43%) complained of pain at the injection site, which disappeared after 1–3 days. No other complications occurred in all the other patients.

**Conclusion:**

The addition of intratympanic or postauricular corticosteroid to systemic steroids did not result in a significant effect on hearing recovery in SSNHL. No obvious complications occur in SSNHL patients treated with intratympanic injection or postauricular injection of corticosteroid.

**Clinical trial registration:**

[chictr.org.cn], registration number: ChiCTR2100048762.

## Introduction

Sudden sensorineural hearing loss (SSNHL) is an idiopathic emergency disease. The recommended treatments for SSNHL do not target the etiology of SSNHL specifically. As a result, a large number of patients cannot be cured completely despite comprehensive treatments. Therefore, it is an urgent challenge for clinicians to improve the treatment efficacy of SSNHL.

The adopted treatments of SSNHL by clinicians include systemic and local application of corticosteroids, vasodilators, defibrinogenating agents, thrombolytics, neurotrophic drugs, antioxidants, antivirals, and hyperbaric oxygen therapy. Currently, the widely accepted effective treatments are systemic and local use of corticosteroids, which are recommended by the latest Chinese and American SSNHL diagnosis and treatment guidelines (1, 2). The pharmacological mechanism of corticosteroids in the treatment of SSNHL has not been fully clarified, including systemic and local effects. The systemic effect is a systemic immunosuppressive response. Regarding local effects, the glucocorticoid exerts effects by combining receptors in the inner ear. These local effects of glucocorticoid include maintaining ion homeostasis in the inner ear, antioxidation, inhibiting apoptosis, downregulating local pro-inflammatory cytokines, and increasing cochlear blood flow (3).

Intratympanic corticosteroid injection for treating SSNHL was used for the first time in 1996 by Silverstein et al. (4). Since then, many clinicians have used this technology. The corticosteroids of the tympanic cavity can penetrate into the inner ear through the round window membrane. This mechanism has been confirmed in many animal experiments which showed that this technology can produce higher drug concentration in the perilymph than intravenous or oral administration (5, 6). After intratympanic injection, the corticosteroids are mainly distributed in the spiral ligament, basement membrane, Organ of Corti, and spiral ganglion (7). A prospective randomized controlled study conducted by Rauch et al. revealed that the effect of tympanic injection of corticosteroid and systemic medication is equivalent (8). Moreover, narrative and systematic reviews showed that there was a lack of a high-quality study to confirm the effectiveness of intratympanic corticosteroid injection for treating SSNHL (9, 10).

Although the effects of corticosteroid tympanic injection alone for treating SSNHL are controversial, there is some evidence that this technology could be used as a salvage treatment for SSNHL patients whose hearing do not restore after 14-day systematic treatment (11, 12). Therefore, intratympanic corticosteroid therapy is recommended by the SSNHL guidelines both in China and the United States as the salvage treatment for SSNHL. In addition to intratympanic corticosteroid treatment, postauricular subperiosteal corticosteroid injection is recommended by the latest Chinese guideline as a salvage procedure. At present, the mechanism of the drug entering the inner ear using this technology is not entirely clear and the speculated routes include circulation and local penetration routes (13). It is presumed that corticosteroids could be absorbed into the circulation *via* postauricular capillaries and lymphatic capillaries and transported to the inner ear *via* its arterial supply, and may also be transported to the inner ear through the bone suture of the auditory vesicle. The drug enters the outer lymph and consequently forms the osmotic gradient between the endolymph and perilymph to exert effects. Currently, the postauricular subperiosteal corticosteroid injection procedure is not mentioned in the American guideline. Therefore, further studies with large sample sizes are needed to evaluate the efficacy of this treatment regimen.

Currently, few reports focus on the treatment effect of local combined with systemic corticosteroid therapy for SSNHL, and most studies have focused on the efficacy of local corticosteroid administration as a salvage treatment for treating SSNHL. However, it is difficult for many patients to return to the hospital several times to receive salvage treatment, and they expect to achieve the best curative effect in the shortest time during hospitalization. In order to evaluate the efficacy of this combined treatment regimen as an initial treatment for SSNHL, we conducted this prospective randomized study. All enrolled SSNHL patients were divided into three groups: intratympanic corticosteroid injection combined with a systemic corticosteroid, postauricular subperiosteal corticosteroid injection combined with a systemic corticosteroid, and systemic corticosteroid treatment alone (control group). Since the prognostic factors of SSNHL include age, the interval from onset to treatment, type of audiometric curve, and degree of hearing loss, we first compared the baseline of clinical characteristics and pre-treatment hearing results of the patients. Then, we compared the hearing efficacy between the patients of the three groups with different audiometric-type SSNHL.

## Materials and methods

### Patients

In this randomized controlled study, we enrolled SSNHL patients hospitalized in the Second Affiliated Hospital of Nanchang University from January 2020 to June 2021. All patients underwent routine physical examination, general otorhinolaryngological examination, nervous system physical examination, pure tone audiogram and tympanometry, laboratory examination, and brain magnetic resonance imaging (MRI) examination.

The study was performed in accordance with the ethical principles and approved by the Second Affiliated Hospital of Nanchang University Institutional Review Board. Written informed consent was obtained from all patients and/or their guardians.

### Inclusion and exclusion criteria

#### Inclusion criteria

Inclusion criteria were as follows: (1) A diagnosis of unilateral SSNH. The diagnostic criteria were based on the latest guidelines revised by the American Academy of Otolaryngology-Head and Neck Surgery in 2019 (2). (2) The interval from onset to treatment was < 1 month.

#### Exclusion criteria

Exclusion criteria were as follows: (1) patients with hearing loss due to other causes such as otitis media, Meniere's disease, otosclerosis, congenital deafness, presbycusis, vestibular schwannoma, and inner ear malformation. (2) The interval from onset to treatment was more than 1 month. (3) Patients who did not undergo standard treatment for 14 days and were discharged without restoring to normal hearing. (4) Patients who had previously received other treatment. (5) Patients with bilateral SSNHL. (6) Patients with contraindications of systemic corticosteroids, such as diabetes, gastrointestinal ulcers, mental disorders, and epilepsy. (7) Patients who dropped out of the study or were lost in follow-up. (8) Patients with insufficient medical record data were also excluded.

**Figure 1:**
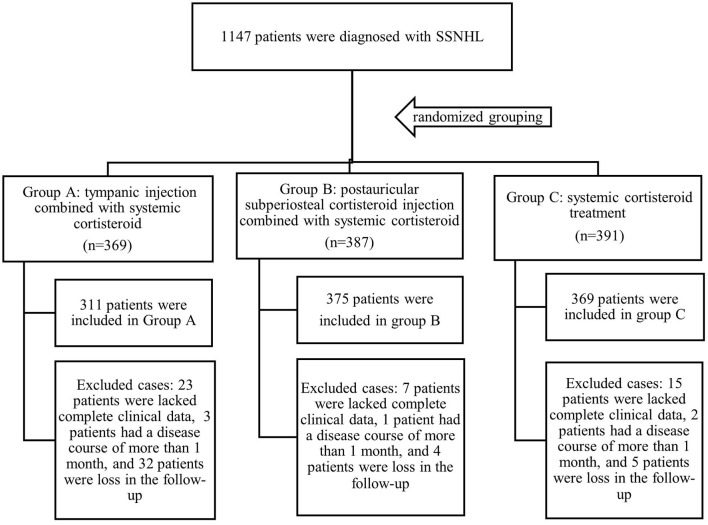
Flow gram of the study.

Patients who met the inclusion criteria were randomly divided into three groups. The patients of group A underwent intratympanic corticosteroid injection combined with systemic corticosteroid treatment; patients of Group B received postauricular subperiosteal corticosteroid injection combined with systemic corticosteroid treatment; and patients of group C were treated only with systemic corticosteroid. Due to the loss of follow-up or lack of complete clinical data, 311, 375, and 369 patients were included in groups A, B, and C, respectively (Figure 1).

### Test procedure

All patients underwent a detailed clinical interview. Clinical data, demographic information, past medical history, and personal history were obtained. Routine physical examination, otolaryngology examination, and audiological and laboratory tests were conducted in all subjects. MRI scanning of the ear and the brain was performed in all patients.

### Treatment procedure

All patients received standard 14-day systemic treatment, which was based on the treatment recommended by the Chinese guidelines for SSNHL diagnosis and treatment revised in 2015 (1). All patients were prescribed 1 mg/kg of prednisone orally per day (maximum dose = 60 mg) for 3 days and were retested audiologically after that. If the treatment was effective, the patients continued to take prednisone for 2 more days; if no effect was seen on the audiogram, the treatment was discontinued on the fourth day. This treatment scheme was in accordance with the latest Chinese guidelines (1), which are revised on the basis of the 2010 German guidelines (14). This guideline recommended that SSNHL patients take prednisone for 3 days, with a total dose of 250 mg. Other medications included antioxidants, neurotrophic, and defibrinogenating agents. The treatment procedure is presented in Table 1.

**Table 1:** Protocol of SSNHL treatment.

**Drugs**	**Treatment procedure**
Prednisone	1 mg per kilogram of body weight (maximum dose 60 mg), orally/3 days. If it was effective, prednisone was continued to be taken for 2 days; if not effective, prednisone was stopped to be taken in the 4th day. Is this treatment according to American and Chinese guidelines? (yes)
Ginkgo biloba extract	105 mg, intravenously/14 days
Vitamin B1	10 mg, orally/14 days
Mecobalamin tablets	500 ug, orally/14 days
Mannitol	Only for patients with ascending-type hearing loss, 50 g, intravenously/14 days
Batroxobin	Only for patients with flat-type and profound hearing loss, 10 BU intravenously for the 1st day, when serum fibrinogen rises to over 1 g/L, intravenous infusion of 5 BU batroxobin again

**Figure 2:**
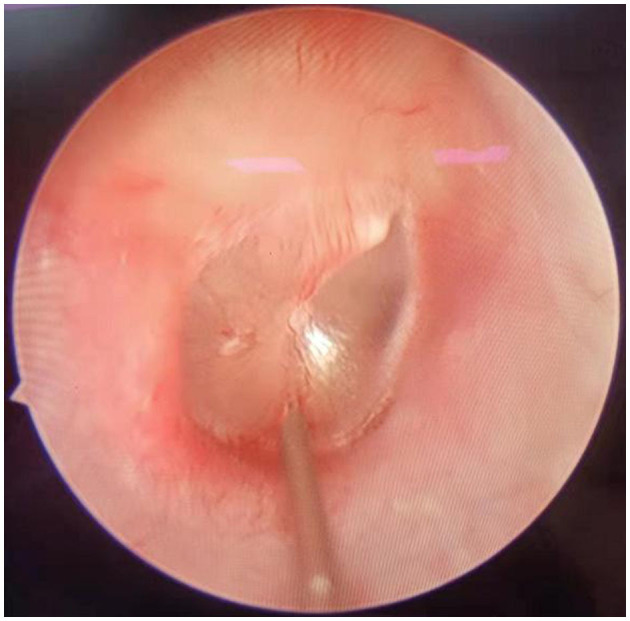
Intratympanic dexamethasone injection for SSNHL patient.

Patients of group A underwent intratympanic dexamethasone injection under otoendoscopy. The procedure was as follows: the patient lay in the lateral position with the ear to be injected upward. After local anesthesia with 1% tetracaine, 0.5 ml of physiological saline and 5 mg of dexamethasone were injected into the middle ear after puncture of the anteroinferior or posteroinferior part of the tympanic membrane (Figure 2). Then, the patient remained still for ~30 min after injection. This procedure was conducted on the first day of treatment and then once every 2 days, a total of four times.

**Figure 3:**
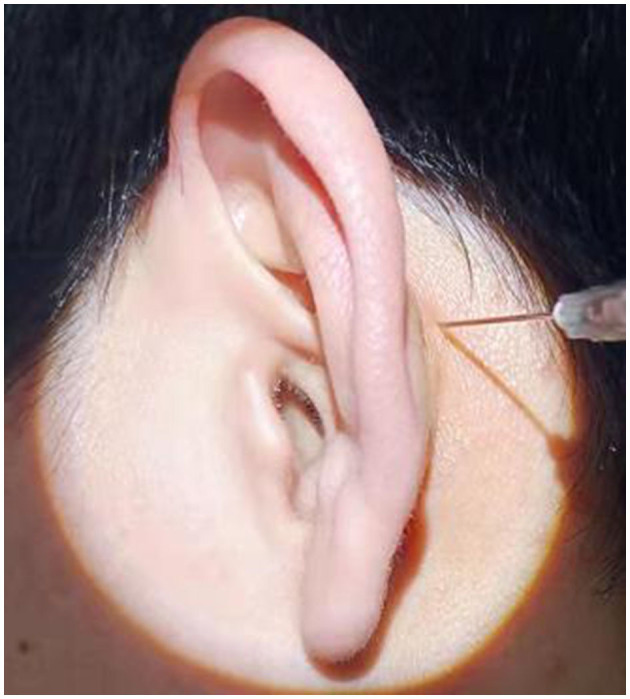
Postauricular subperiosteal methylprednisolone injection for SSNHL patient.

In group B, postauricular subperiosteal methylprednisolone injection was performed in all patients. The procedure was as follows: the patient sat on the chair. After the postauricular skin was disinfected, 40 mg (1 ml) of methylprednisolone was injected at the postauricular site. The injection site was located 0.5 cm behind the posterior sulcus of the affected ear and was level with the posterosuperior part of the external auditory meatus (Figure 3). After the injection, the injection point was compressed for 5 min. This procedure was performed on the first day of treatment and then once every 2 days, a total of four times.

All patients' hearing was evaluated with pure tone audiogram and tympanometry. All hearing tests were carried out by the same audiologist. Air and bone conduction was assessed at frequencies of 250 Hz, 500 Hz, 1 kHz, 2 kHz, 4 kHz, and 8 kHz.

Pure tone audiogram (PTA) was calculated by averaging air conduction thresholds at 0.5, 1, 2, and 4 kHz (15). The hearing loss levels were categorized into four grades: mild (26–40 dB HL), moderate (41–55 dB HL), moderate to severe (56–70 dB HL), severe (71–90 dB HL), and profound (>90 dB HL) (15). Audiogram patterns were classified into five types: ascending (the average threshold of 0.25–0.50 kHz was 20 dB higher than that of 4–8 kHz), descending (the average threshold of 4–8 kHz was 20 dB higher than that of 0.25–0.50 kHz), flat (all frequencies present similar thresholds and the hearing threshold was below 80 dB HL), profound (all frequencies show similar threshold and the hearing threshold was over 80 dB HL), and concave or convex type (average hearing degree of the mid-tone frequency was 20 dB higher than low and high frequencies) (1).

In addition to calculating patients' PTA, we evaluated patients' hearing by calculating the average air conduction hearing threshold at hearing impairment frequency. The patients' hearing of the affected ear was assessed by referring to the unaffected ears' hearing or their affected ears' initial hearing. Referring to the 2015 Chinese guidelines (1), the average air conduction hearing threshold at hearing impairment frequency was calculated as follows: among patients with flat-type or profound hearing loss, the hearing threshold at hearing impairment frequency was equivalent to the average hearing threshold of all frequencies. For the patients with low-frequency or high-frequency hearing loss, it was calculated as the average hearing threshold at hearing impairment low or high frequencies.

With reference to the Chinese guidelines for the diagnosis and treatment of SSNHL revised in 2015 (1), by comparing the hearing results before and 6 months after treatment, the hearing recovery of all patients was categorized into four grades: complete recovery, effective, significantly effective, and ineffective, as shown in Table 2.

**Table 2:** Classification and standard of hearing efficacy.

**Classification**	**Standard**
Complete recovery	The hearing threshold at hearing impairment frequency is within normal limits, or reached to the hearing level of unaffected ear, or reached to the hearing level of affected ear's initial hearing
Significantly effective	>30 dB HL improvement at hearing impairment frequency
Effective	15- to 30 dB HL improvement at hearing impairment frequency
Ineffective	< 15 dB HL improvement at hearing impairment frequency

### Follow-up procedure

We tested the patients' hearing on the seventh day and 1 day before discharge after treatment, and retest their hearing immediately as long as they reported hearing improvement during hospitalization. If their hearing recovered, the treatment was terminated. If their hearing did not recover to normal, they would be instructed to continue taking 7.5 mg of *Ginkgo biloba* extract, 10 mg of vitamin B1, and 0.5 mg of Mecobalamin orally three times a day for 30 days. The second time of hearing examination was 30 days after discharge. If their hearing completely reached normal, drugs would be discontinued. If their hearing did not recover completely, the same cure would be given for 2 months. Three months after discharge, all treatments were terminated and patients revisited our hospital. The last follow-up time was 6 months after treatment, and their hearing was reevaluated and was taken as the final hearing result to evaluate the hearing effect. During the follow-up period, in addition to evaluating the patient's hearing, the general otorhinolaryngological examination and otoendoscopy were performed at each follow-up visit.

### Statistical analysis

Quantitative data were presented as mean ± standard deviation for comparison, while frequency data were presented as cases and the ratio for comparison. An analysis of variance (ANOVA) was performed for the data conforming to normal distribution and homogeneity, while a Kruskal–Wallis test was conducted for those not conforming to normal distribution or homogeneity. Categorical data were shown as percentages and compared using the chi-square test. The Fisher exact test was used when expected counts in the chi-square test were insufficient. All analyses were conducted using SPSS version 25 for Windows. All statistical tests were two-sided, and statistically significant levels were set at a *P*-value of 0.05.

## Result

### Clinical characteristics and pre- and post-treatment hearing results of the patients in the three groups

Table 3 shows the clinical characteristics and post-treatment hearing results of the patients in the three groups. The case number of groups A, B, and C was 311, 375, and 369, respectively. The mean age of the patients in the three groups was 46.35 ± 15.104, 44.79 ± 13.893, and 41.85 ± 16.185 years. The number of male patients in the three groups was 151, 211, and 272, and the number of female patients in the three groups was 160, 164, and 197, respectively. There was a significant difference in the average ages of the three groups (*P* < 0.01). However, no significant difference existed in patients' gender distribution, the proportion of affected left or right ears, and the average interval from onset to treatment between the three groups (*P* > 0.05).

**Table 3:** Clinical characteristics and pre- and post-treatment hearing of patients of the three groups.

**Characteristics**	**Group A (*n* = 311)**	**Group B (*n* = 375)**	**Group C (*n* = 369)**	**Statistical values**	***P*-values**
Age (years), mean ± standard deviation	46.35 ± 15.104	44.79 ± 13.893	41.85 ± 16.185	−4.915	0.000^※^
**Gender**					
Male [cases (%)]	151 (48.6)	211 (56.3)	172 (46.6)	7.686	0.021^&^
Female [cases (%)]	160 (51.4)	164 (43.7)	197 (53.4)		
**Side of affected ear**					
Left [cases (%)]	149 (47.9)	194 (51.7)	189 (51.2)	1.137	0.566^&^
Right [cases (%)]	162 (52.1)	181 (48.3)	180 (48.8)		
Interval from onset to treatment (days), mean ± standard deviation	7.59 ± 7.104	5.076 ± 5.139	7.07 ± 7.014	−0.577	0.564^※^
**Type of auditory curve before treatment**					
Ascending type [cases (%)]	18 (5.8)	53 (14.1)	135 (36.6)	172.677	0.000^&^
Descending type [cases (%)]	14 (4.5)	16 (4.3)	21 (5.7)		
Flat type [cases (%)]	175 (56.3)	117 (31.2)	144 (39)		
Profound type [cases (%)]	104 (33.4)	189 (50.4)	69 (18.7)		
**Degree of hearing loss before treatment**					
Mild [cases (%)]	17 (5.5)	20 (5.3)	107 (29)	164.308	0.000^&^
Moderate [cases (%)]	57 (18.3)	53 (14.1)	99 (26.8)		
Moderate to severe [cases (%)]	65 (20.9)	63 (16.8)	52 (14.1)		
Severe [cases (%)]	69 (22.2)	98 (26.1)	50 (13.6)		
Profound [cases (%)]	103 (33.1)	141 (37.6)	61 (16.5)		
**Pre- and post-treatment hearing results**					
Pre-treatment PTA (dB, HL)	76.998 ± 28.708	79.194 ± 30.428	53.677 ± 33.309	134.486	0.000^※^
Post-treatment PTA (dB, HL)	57.727 ± 31.177	56.689 ± 34.151	38.692 ± 31.134	92.206	0.000^※^
PTA gap (dB, HL)	19.272 ± 21.420	22.505 ± 22.893	14.985 ± 17.334	17.715	0.000^※^
Pre-treatment average hearing threshold at hearing impairment frequency (dB, HL)	78.302 ± 25.706	81.885 ± 25.983	61.132 ± 27.403	64.503	0.000^#^
Post-treatment average hearing threshold at hearing impairment frequency (dB, HL)	59.097 ± 29.614	58.405 ± 32.576	42.625 ± 29.490	74.846	0.000^※^
Hearing gap (dB, HL)	19.205 ± 20.115	23.479 ± 21.740	18.507 ± 16.681	10.207	0.006^※^
**Hearing efficacy**					
Complete recovery [cases (%)]	46 (14.8)	85 (22.7)	165 (44.6)	94.127	0.000^&^
Significant effective [cases (%)]	53 (17)	85 (22.7)	40 (10.8)		
Effective [cases (%)]	65 (20.9)	62 (16.5)	42 (11.4)		
Ineffective [cases (%)]	147 (47.3)	143 (38.1)	123 (33.2)		

^#^Variance Analysis.

^&^Chi-square test.

^※^Kruskal–Wallis test.

**Figure 4:**
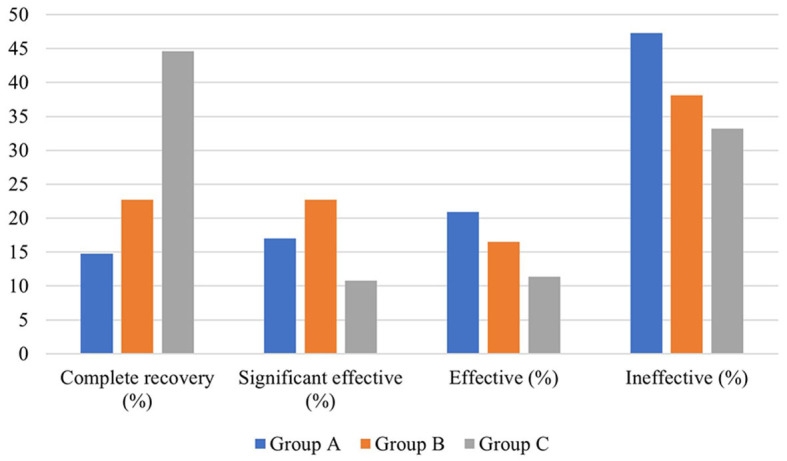
Hearing efficacy of SSNHL patients in the three groups.

In terms of hearing results, a significant difference existed in the distribution of pre-treatment auditory curve type and degree of hearing loss among the three groups (*P* < 0.01). The pre- and post-treatment PTA and the PTA gap of the patients in the three groups were also significantly different (*P* < 0.05). The biggest pre- and post-treatment PTA gap existed in group B, followed by group A and group C. In addition, the pre- and post-treatment average hearing thresholds at the impairment frequency in patients of the three groups were significantly different (*P* < 0.05). As for the pre- and post-treatment gap of hearing thresholds at hearing impairment frequency, the largest one existed in group B (23.479 ± 21.740 dB HL), followed by group A (19.205 ± 20.115 dB, HL) and group C (18.507 ± 16.681 dB, HL). This gap was statistically significant (*P* < 0.05). In terms of grading of hearing efficacy, the highest one was in patients of group C (66.8%), followed by group B (61.9%) and group A (52.7%) (Figure 4).

### Efficacy of different topical corticosteroid administration methods in patients with different auditory types

The baseline of patients in the three groups including the average age, gender distribution, and the distribution of auditory types and hearing degrees was inequivalent, which may affect the post-treatment hearing effect. Therefore, we analyzed the efficacy of SSNHL patients with different audiogram-type SSNHL.

### Efficacy of different local corticosteroids combined with systemic corticosteroid administration in patients with ascending-type SSNHL

**Figure 5:**
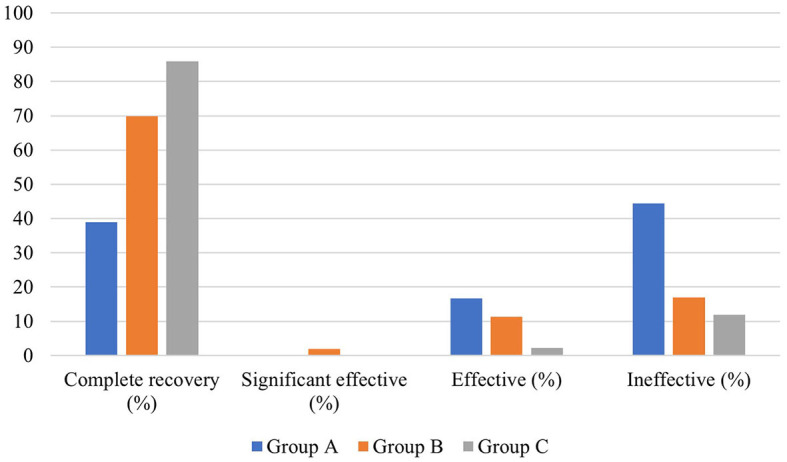
Hearing efficacy of patients with ascending-type SSNHL in the three groups.

There was no significant difference in average age, gender distribution, and average interval from onset to treatment among patients in the three groups (*P* > 0.05). In terms of hearing results, a significant difference existed in the pre- and post-treatment average hearing threshold at the hearing impairment frequency among the patients in the three groups (*P* < 0.05), but no significant difference was observed in the gap between pre- and post-treatment average hearing threshold at the hearing impairment frequency (*P* > 0.05), which were 17.454 ± 15.166 dB, 22.296 ± 14.959 dB, and 18.91 ± 13.142 dB, respectively. However, there was a significant difference in the proportion of patients with different hearing efficacy grading in the three groups (*P* < 0.05). Furthermore, the hearing recovery rate of patients in group C was higher than those in the other two groups (Table 4, Figure 5).

**Table 4:** Clinical characteristics and hearing efficacy of patients with ascending-type SSNHL in the three groups.

**Characteristics**	**Group A (*n* = 18)**	**Group B (*n* = 53)**	**Group C (*n* = 135)**	**Statistical values**	***P*-values**
**Age (years), mean** **±standard deviation**	41.39 ± 12.363	39.45 ± 11.533	38.23 ± 12.090	4.041	0.07^#^
Male [cases (%)]	6 (33.3)	28 (52.8)	46 (57.5)	5.888	0.053^&^
Female [cases (%)]	12 (66.7)	25 (47.2)	89 (70.6)		
Interval from onset to treatment (days), mean ± standard deviation	10.833 ± 8.932	8.953 ± 4.081	8.222 ± 6.180	9.159	0.1^※^
**Degree of hearing loss before treatment**					
Mild [cases (%)]	3 (16.7)	12 (22.6)	69 (51.1)	27.687	0.000^@^
Moderate [cases (%)]	12 (66.7)	28 (52.8)	59 (43.7)		
Moderate to severe [cases (%)]	3 (16.7)	10 (18.9)	7 (5.2)		
Severe [cases (%)]	0 (0)	3 (5.7)	0 (0)		
Profound [cases (%)]	0 (0)	0 (0)	0 (0)		
**Pre- and post-treatment hearing results**					
Pre-treatment average hearing threshold at hearing impairment frequency (dB, HL)	49.097 ± 7.983	49.048 ± 10.270	41.744 ± 8.122	16.407	0.000^#^
Post-treatment average hearing threshold at hearing impairment frequency (dB, HL)	31.643 ± 14.759	26.753 ± 14.114	22.834 ± 12.596	4.481	0.012^#^
Hearing gap (dB, HL)	17.454 ± 15.166	22.296 ± 14.959	18.91 ± 13.142	1.395	0.25^#^
**Hearing efficacy**					
Completely recovery [cases (%)]	7 (38.9)	37 (69.8)	116 (85.9)	26.365	0.000^@^
Significantly effective [cases (%)]	0 (0)	1 (1.9)	0 (0)		
Effective [cases (%)]	3 (16.7)	6 (11.3)	3 (2.2)		
Ineffective [cases (%)]	8 (44.4)	9 (17)	16 (11.9)		

^#^Variance Analysis.

^&^Chi-square test.

^※^Kruskal–Wallis test.

^@^Fisher exact test.

### Efficacy of different local glucocorticoids combined with systemic glucocorticoid administration in patients with descending-type SSNHL

**Figure 6:**
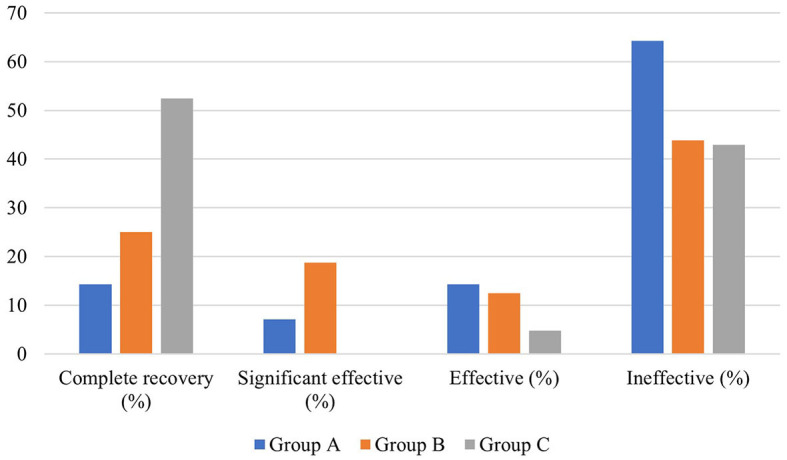
Hearing efficacy of patients with descending-type SSNHL in the three groups.

There was no significant difference in the average age, gender distribution, average interval from onset to treatment, and distribution of pre-treatment hearing loss degree among patients in the three groups (*P* > 0.05). In addition, no significant difference existed in the pre- and post-treatment average hearing threshold at the hearing impairment frequency among patients of the three groups (*P* > 0.05), as well as in the gap of the pre- and post-treatment average hearing threshold at the hearing impairment frequency between them (*P* > 0.05). Similarly, there was no significant difference in the proportion of patients with different hearing efficacy grading among patients in the three groups (*P* > 0.05) (Table 5, Figure 6).

**Table 5:** Clinical characteristics and hearing efficacy of patients with descending-type SSNHL in the three groups.

**Characteristics**	**Group A (*n* = 14)**	**Group B (*n* = 16)**	**Group C (*n* = 21)**	**Statistical values**	***P*-values**
**Age (years), mean** **±standard deviation**	43.5 ± 13.794	36.44 ± 3.306	34.24 ± 15.109	1.845	0.169^#^
Male [cases (%)]	9 (64.3)	9 (56.3)	13 (60.8)	0.221	0.895^&^
Female [cases (%)]	5 (35.7)	7 (43.8)	8 (38.1)		
Interval from onset to treatment (days), mean ± standard deviation	9.93 ± 10.011	6.19 ± 7.185	8.71 ± 8.684	0.75	0.478^#^
**Degree of hearing loss before treatment**					
Mild [cases (%)]	0 (0)	1 (6.3)	1 (4.8)	6.57	0.6^@^
Moderate [cases (%)]	5 (35.7)	3 (18.8)	7 (33.3)		
Moderate to severe [cases (%)]	4 (28.6)	3 (18.8)	6 (28.6)		
Severe [cases (%)]	1 (7.1)	5 (31.3)	5 (23.8)		
Profound [cases (%)]	4 (28.6)	4 (25)	2 (9.5)		
**Pre- and post-treatment hearing results**					
Pre-treatment average hearing threshold at hearing impairment frequency (dB, HL)	68.095 ± 19.575	74.792 ± 22.679	66.111 ± 20.817	0.804	0.453^#^
Post-treatment average hearing threshold at hearing impairment frequency (dB, HL)	53.452 ± 27.563	55.547 ± 21.703	52.738 ± 27.846	0.55	0.947^#^
Hearing gap (dB, HL)	14.642 ± 19.814	19.244 ± 20.249	13.373 ± 11.348	0.396	0.82^※^
**Hearing efficacy**					
Completely recovery [cases (%)]	2 (14.3)	4 (25)	11 (52.4)	9.504	0.098^@^
Significantly effective [cases (%)]	1 (7.1)	3 (18.8)	0 (0)		
Effective [cases (%)]	2 (14.3)	2 (12.5)	1 (4.8)		
Ineffective [cases (%)]	9 (64.3)	7 (43.8)	9 (42.9)		

^#^Variance Analysis.

^&^Chi-square test.

^※^Kruskal–Wallis test.

^@^Fisher exact test.

### Efficacy of different local corticosteroids combined with systemic corticosteroid administration in patients with flat-type SSNHL

**Figure 7:**
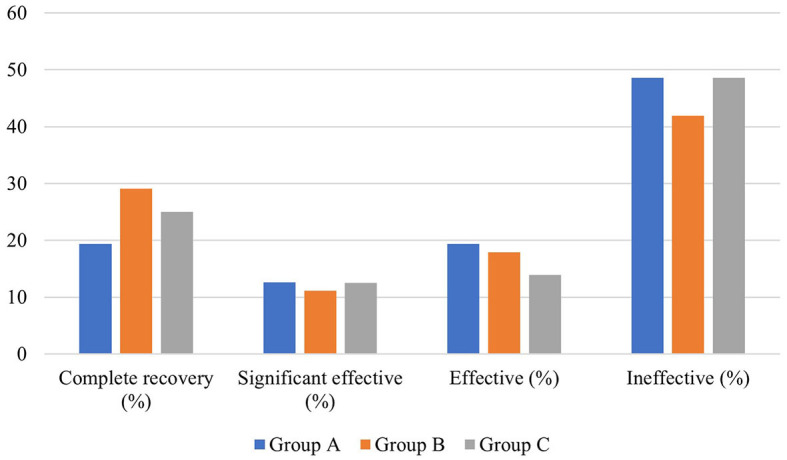
Hearing efficacy of patients with flat-type SSNHL in the three groups.

There was no significant difference in average age, gender distribution, and average interval from onset to treatment among patients in the three groups (*P* > 0.05), but there was a significant difference in the degree of hearing loss before treatment and pre- and post-treatment average hearing threshold at the hearing impairment frequency among patients in the three groups (*P* < 0.05). However, no significant difference was observed in the gap between the pre- and post-treatment average hearing threshold at the hearing impairment frequency (*P* > 0.05). Moreover, there was no significant difference in the proportion of patients with different hearing efficacy grading among patients in the three groups (*P* > 0.05) (Table 6, Figure 7).

**Table 6:** Clinical characteristics and hearing efficacy of patients with flat-type SSNHL in the three groups.

**Characteristics**	**Group A (*n* = 175)**	**Group B (*n* = 117)**	**Group C (*n* = 144)**	**Statistical values**	***P*-values**
**Age (years), mean** **±standard deviation**	47.99 ± 14.637	46.32 ± 13.927	45.76 ± 15.854	0.976	0.378^#^
**Gender**					
Male [cases (%)]	85 (48.6)	68 (58.1)	82 (56.9)	3.375	0.185^&^
Female [cases (%)]	90 (51.4)	49 (41.9)	62 (43.1)		
Interval from onset to treatment (days), mean ± standard deviation	7.897 ± 6.8	5.56 ± 5.000	7.167 ± 7.318	4.697	0.095^※^
**Degree of hearing loss before treatment**					
Mild [cases (%)]	14 (8)	7 (6.0)	37 (25.7)	32.261	0.000^@^
Moderate [cases (%)]	40 (22.9)	23 (19.7)	33 (22.9)		
Moderate to severe [cases (%)]	58 (33.1)	49 (141.9)	39 (27.1)		
Severe [cases (%)]	61 (34.9)	38 (32.5)	35 (24.3)		
Profound [cases (%)]	2 (1.1)	0 (0)	0 (0)		
**Pre- and post-treatment hearing results**					
Pre-treatment average hearing threshold at hearing impairment frequency (dB, HL)	64.237 ± 15.242	63.511 ± 13.458	56.266 ± 17.128	18.492	0.000^※^
Post-treatment average hearing threshold at hearing impairment frequency (dB, HL)	46.559 ± 20.059	42.652 ± 20.367	39.683 ± 18.676	4.884	0.008^#^
Hearing gap (dB, HL)	17.679 ± 19.517	20.859 ± 19.253	16.583 ± 16.410	4.097	0.129^※^
**Hearing efficacy**					
Completely recovery [cases (%)]	34 (19.4)	34 (29.1)	36 (25)	5.274	0.509^&^
Significantly effective [cases (%)]	22 (12.6)	13 (11.1)	18 (12.5)		
Effective [cases (%)]	34 (19.4)	21 (17.9)	20 (13.9)		
Ineffective [cases (%)]	85 (48.6)	49 (41.9)	70 (48.6)		

^#^Variance Analysis.

^&^Chi-square test.

^※^Kruskal–Wallis test.

^@^Fisher exact test.

### Efficacy of different local corticosteroids combined with systemic corticosteroid administration in patients with profound SSNHL

**Figure 8:**
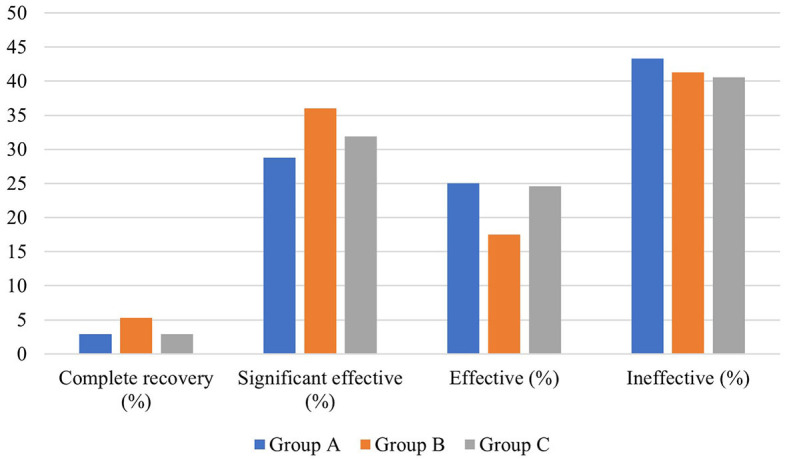
Hearing efficacy of patients with profound SSNHL in the three groups.

There was no significant difference in average age, gender distribution, and average interval from onset to treatment among patients in the three groups (*P* > 0.05). Regarding the hearing result, there was a significant difference in the pre-treatment average hearing threshold at the hearing impairment frequency (*P* < 0.05), but no significant difference in the post-treatment average hearing threshold at the hearing impairment frequency, as well as the pre- and post-treatment gap at the hearing impairment frequency between the three groups (*P* > 0.05). Moreover, there was no significant difference in the proportion of patients with different hearing efficacy grading among patients in the three groups (*P* > 0.05) (Table 7, Figure 8).

**Table 7:** Clinical characteristics and hearing efficacy of patients with profound SSNHL in the three groups.

**Characteristics**	**Group A (*n* = 104)**	**Group B (*n* = 189)**	**Group C (*n* = 69)**	**Statistical values**	***P*-values**
Age (years), mean ± standard deviation	47.36 ± 16.327	46.71 ± 13.618	51.86 ± 15.855	6.413	0.052^※^
**Gender**					
Male [cases (%)]	52 (50)	106 (56.1)	31 (44.9)	2.807	0.246^&^
Female [cases (%)]	52 (50)	83 (43.9)	38 (55.1)		
Interval from onset to treatment (days), mean ± standard deviation	6.365 ± 6.614	7.719 ± 5.292	8.014 ± 7.279	10.593	0.061^※^
**Pre- and post-treatment hearing results**					
Pre-treatment average hearing threshold at hearing impairment frequency (dB, HL)	108.397 ± 11.119	102.979 ± 14.181	107.705 ± 13.579	12.434	0.002^※^
Post-treatment average hearing threshold at hearing impairment frequency (dB, HL)	85.705 ± 26.645	77.302 ± 31.060	84.408 ± 28.868	5.727	0.057^※^
Hearing gap (dB, HL)	22.692 ± 21.616	25.676 ± 24.680	23.366 ± 23.468	0.629	0.534^#^
**Hearing efficacy**					
Completely recovery [cases (%)]	3 (2.9)	10 (5.3)	2 (2.9)	4.773	0.573^&^
Significantly effective [cases (%)]	30 (28.8)	68 (36)	22 (31.9)		
Effective [cases (%)]	26 (25)	33 (17.5)	17 (24.6)		
Ineffective [cases (%)]	45 (43.3)	78 (41.3)	28 (40.6)		

^#^Variance Analysis.

^&^Chi-square test.

^※^Kruskal–Wallis test.

^@^Fisher exact test.

### Complications and follow-up results

In group A, 33 patients (10.6%) had a transient vertigo attack during tympanic injection, which lasted for ~1–3 min; 20 patients felt earache, lasting for 1–3 days. In group B, 18 patients (4.8%) complained of swelling and pain at the injection site, which disappeared within 2 days. No obvious complications such as tympanic membrane perforation or acute and chronic otitis media occurred in patients of group A during follow-up. In addition, there were no complications in all other patients during follow-up.

## Discussion

The possible prognostic factors of SSNHL include the patient's age, the audiogram type, the degree of hearing loss, and the interval from onset to treatment (1, 16). The cofactors including the average age, gender distribution, and the distribution of auditory types and hearing degrees in the three groups were inequivalent, so we analyzed the efficacy of SSNHL patients with different audiogram-type SSNHL. It is reported that comparing patients with other types of audiometric curves, patients with low-frequency hearing loss may have a better prognosis (1, 17). In 2015, a Chinese multicenter study showed that the curative rate of patients with low-frequency hearing loss could be as high as 90.73%, whereas, in patients with high-frequency hearing loss, the number was only 65.96% (18).

The pathogenesis of SSNHL with various audiogram types may be different, which may explain this discrepancy. For example, the proposed pathogenesis of low-frequency hearing loss may be inner ear hydrops; the high-frequency hearing loss may be due to hair cell injury; the flat-type hearing loss is mostly caused by stria vascularis dysfunction or inner ear vasospasm; and the etiology of profound hearing loss is inner ear vascular embolism or thrombosis (14).

The effectiveness of intratympanic injection combined with systemic corticosteroids for treating SSNHL is still controversial. Several researchers showed that the effect of intratympanic combined with systemic corticosteroid therapy was better than that of systemic corticosteroid administration alone (19, 20). For example, a recent meta-analysis study result showed that the treatment effects ranked from high to low were as follows: intravenous, intratympanic corticosteroid, intravenous combined with an oral corticosteroid, intratympanic corticosteroid, intravenous corticosteroid, oral corticosteroid, and placebo (19). The results of animal experiments demonstrated that through intratympanic corticosteroid route, more drugs could be delivered into the inner ear, longer therapeutic window and more effective result could be achieved than by intravenous or intratympanic injection route alone in the pharmacokinetics (21). Therefore, researchers concluded that the curative effect of the combination of corticosteroid might be better than that of intravenous or intratympanic corticosteroid therapy alone. Indeed, theoretically, a blood–labyrinth barrier may limit the therapeutic agents permeating into the inner ear, and combined corticosteroid administration may reach higher drug concentrations and better effects. However, a prospective randomized study showed that the recovery rates in the patients with combined treatment and intratympanic injection treatment were 70 and 73%, respectively, and no significant difference existed between the patients of the two groups (3). In addition, another systematic meta-analysis also confirmed that the effect of combined therapy was equivalent to that of systemic or intratympanic injection of corticosteroid alone, and the hearing effect of intratympanic injection was similar to systemic corticosteroid among patients with moderate and severe SSNHL (22). Furthermore, our study results demonstrated that there was no significant difference between the efficacy of intratympanic injection combined with systemic corticosteroid treatment and the control group, suggesting that systemic corticosteroid treatment can achieve a similar efficacy as combined treatment as an initial treatment. We speculate that the reason for this phenomenon is that inner ear hormone receptors are saturated after patients receive a sufficient dose of systemic corticosteroid treatment, so the additional local medication has no additional effect.

Intratympanic corticosteroid injection has its own advantages. The first one is that it can avoid the side effects of systemic corticosteroid administration, which is contraindicated in patients with diabetes, hypertension, tumor, infection, and acute stage of peptic ulcer. Second, intratympanic injection is easy to operate. However, this technology is an invasive operation and may result in some local complications, such as pain, dizziness, and secondary acute or chronic otitis media. Therefore, it is necessary to select treatment strategies according to the patient's own circumstances. For example, for patients with contraindications of systemic administration of corticosteroids, local administration of corticosteroids can be taken as a priority.

Regarding the type of corticosteroid selected for intratympanic injection, dexamethasone, methylprednisolone, and prednisone are most used by medical institutions. Animal experiment results demonstrated that methylprednisolone has the highest permeability after intratympanic injection compared with dexamethasone and prednisone (5). However, the degree and incidence of pain response in patients after intratympanic methylprednisolone injection were significantly higher than those of patients who underwent intratympanic dexamethasone injection (23). Before we carried out this study, we also initially applied intratympanic methylprednisolone injection for SSNHL patients and found that many patients complained of unbearable earache. Some patients even suffered from tympanic perforation and ear pus. In contrast, a recent meta-analysis study demonstrated that as a salvage treatment for SSNHL, intratympanic injection of dexamethasone was more effective than methylprednisolone (24). Therefore, we chose dexamethasone as the intratympanic injection agent for treating SSNHL. Referring to the therapeutic schedule reported in the previous literature and recommended by the guidelines, we performed an intratympanic injection of 5 mg dexamethasone once a day, four times in total for SSNHL patients.

As a new treatment scheme for SSNHL, postauricular corticosteroid injection has been more and more valued by otologists. This scheme is recommended by the 2015 Chinese guideline for the diagnosis and treatment of SSNHL (1) but is not widely applied worldwide. Both animal and clinical studies have verified the effectiveness of the postauricular injection route. Wang et al. (25) explored the cochlear concentration and distribution of dexamethasone after administration by intratympanic, post-aural, and intraperitoneal methods. They found that intratympanic and post-aural administration could result in higher dexamethasone concentrations in the Organ of Corti than systemic administration, but systemic administration could produce higher dexamethasone concentrations in the stria vascularis than the other administration methods. Li et al. (13) conducted an animal study by using 7.0 Testa magnetic resonance imaging for guinea pigs after postauricular and intravenous injection of gadopentetate dimeglumine (Gd). They measured the relative signal intensity in the scala tympani of the basal turn to evaluate indirectly the dynamic Gd uptake in the perilymph. They found a delayed time to peak enhancement, prolonged elimination half-life, extended mean residence time, and a greater area under the signal–time curve among postauricularly treated guinea pigs. This study's results indicate that the bioavailability of drugs may increase to a certain extent and achieve a better effect through the postauricular injection route than systemic administration. In clinical studies, a retrospective study that enrolled 63 refractory SSNHL patients has revealed that the patients who underwent postauricular corticosteroid administration as a salvage treatment demonstrated better results than untreated patients. The most frequent adverse event was injection pain; other major adverse events included sleep change, increase in blood glucose, and headache (26). Moreover, another multicenter clinical study that enrolled 173 SSNHL patients showed that the clinical efficacy of intratympanic steroid perfusion and postauricular steroid injection was similar for refractory severe and profound SSNHL patients (27). In contrast, compared with intratympanic injection, the postauricular injection has its own advantages. It is simple to operate and less likely to generate complications. However, due to the small number of literature reporting the effect of this technology currently, its effectiveness is uncertain. In future, more studies are needed to further investigate the efficacy and safety of this technology in SSNHL treatment. Although complications might be more likely to occur by applying the intratympanic injection route, our clinical practice shows that this technology is a safe procedure, since no patient suffered from severe complications during the follow-up period. In order to ensure the effectiveness of the medication, for patients with contraindications to systemic medication, intratympanic injection route is still a top priority currently.

The limitation of this study is that after stratification, the sample size of subjects in each subgroup is limited, although our study has a large sample size. Therefore, it is difficult to ensure the equivalence of the baseline such as age or pre-treatment hearing level between each group, which may generate methodological bias and affect the accuracy of the results. Nevertheless, since the baselines of most subgroups are equivalent, our results are convincing. In contrast, in order to ensure the treatment effect, patients received a combination of multiple therapies such as antioxidants, neurotrophic agents, and defibrinogen, which may also result in heterogeneity. In addition, a randomized blind placebo control multicenter study is necessary before we make a clear conclusion.

## Conclusion

The exact role of intratympanic and postauricular corticosteroids requires additional trials. The addition of intratympanic or postauricular corticosteroid to systemic steroids did not result in a significant effect on hearing recovery in SSNHL. However, for patients with contraindications to systemic corticosteroid administration, intratympanic corticosteroid or postauricular corticosteroid may be safe and efficacious alternatives.

## Data availability statement

The raw data supporting the conclusions of this article will be made available by the authors, without undue reservation.

## Ethics statement

The studies involving human participants were reviewed and approved by the Second Affiliated Hospital of Nanchang University Institutional Review Board. Written informed consent to participate in this study was provided by the participants' legal guardian/next of kin.

## Author contributions

MD and YL conceived and designed the study. WX, NK, JL, and HP performed the experiments. CL and ZZ collected data. WX, NK, and MD wrote the manuscript. All authors contributed to the writing and final approval of the manuscript.

## References

[1] Editorial Board of Chinese Journal of Otorhinolaryngology Head and Neck Surgery; Society of Otorhinolaryngology Head and Neck Surgery CMA. Guideline of diagnosis and treatment of sudden deafness (2015). Zhonghua er bi Yan Hou Tou Jing Wai ke Za Zhi. (2015) 50:443–7. 26695792

[2] Chandrasekhar SS Tsai Do BS Schwartz SR Bontempo LJ Faucett EA Finestone SA. Clinical practice guideline: sudden hearing loss (update). Otolaryngol Head Neck Surg. (2019) 161:S1–45. 10.1177/0194599819859885 31369359

[3] Ahn JH Yoo MH Yoon TH Chung JW. Can intratympanic dexamethasone added to systemic steroids improve hearing outcome in patients with sudden deafness? Laryngoscope. (2008) 118:279–82. 10.1097/MLG.0b013e3181585428 17989574

[4] Haynes DS O'Malley M Cohen S Watford K Labadie RF. Intratympanic dexamethasone for sudden sensorineural hearing loss after failure of systemic therapy. Laryngoscope. (2007) 117:3–15. 10.1097/01.mlg.0000245058.11866.15 17202923

[5] Parnes LS Sun AH Freeman DJ. Corticosteroid pharmacokinetics in the inner ear fluids: an animal study followed by clinical application. Laryngoscope. (1999) 109:1–17. 10.1097/00005537-199907001-00001 10399889

[6] Chandrasekhar SS. Intratympanic dexamethasone for sudden sensorineural hearing loss: clinical and laboratory evaluation. Otol Neurotol. (2001) 22:18–23. 10.1097/00129492-200101000-00005 11314710

[7] Hargunani CA Kempton JB DeGagne JM Trune DR. Intratympanic injection of dexamethasone: time course of inner ear distribution and conversion to its active form. Otol Neurotol. (2006) 27:564–9. 10.1097/01.mao.0000194814.07674.4f 16691147

[8] Rauch SD Halpin CF Antonelli PJ Babu S Carey JP Gantz BJ. Oral vs intratympanic corticosteroid therapy for idiopathic sudden sensorineural hearing loss: a randomized trial. JAMA. (2011) 305:2071–9. 10.1001/jama.2011.679 21610239

[9] Doyle KJ Bauch C Battista R Beatty C Hughes GB Mason J. Intratympanic steroid treatment: a review. Otol Neurotol. (2004) 25:1034–9. 10.1097/00129492-200411000-00031 15547441

[10] Hu A Parnes LS. Intratympanic steroids for inner ear disorders: a review. Audiol Neurootol. (2009) 14:373–82. 10.1159/000241894 19923807

[11] Choi JW Lee CK Kim SB Lee DY Ko SC Park KH. Potential benefits of salvage intratympanic dexamethasone injection in profound idiopathic sudden sensorineural hearing loss. Eur Arch Otorhinolaryngol. (2020) 277:2219–27. 10.1007/s00405-020-05967-z 32296977

[12] Amarillo E Hernando M Eisenberg G Granda M Plaza G. Efficacy of intratympanic corticosteroid as a salvage treatment in idiopathic sudden sensorineural hearing loss. Acta Otorrinolaringol Espanola. (2019) 70:207–14. 10.1016/j.otoeng.2018.04.006 30093088

[13] Li J Yu L Xia R Gao F Luo W Jing Y. Postauricular hypodermic injection to treat inner ear disorders: experimental feasibility study using magnetic resonance imaging and pharmacokinetic comparison. J Laryngol Otol. (2013) 127:239–45. 10.1017/S0022215113000017 23406669

[14] Ganzer U Albegger KW Arnold W. Leitlinie ‘Hörsturz'. Konsensusbericht in Auftrag des präsidium der Deutschen Gesellschaft für Hals-Nasen-Ohren-Heikunde, Kopf-und Hals-Chirurgie. HNO Information. (2004) 4:302–8.

[15] Clark JG. Uses and abuses of hearing loss classification. Asha. (1981) 23:493–500. 7052898

[16] Kang WS Yang CJ Shim M Song CI Kim TS Lim HW. Prognostic factors for recovery from sudden sensorineural hearing loss: a retrospective study. J Audiol Otol. (2017) 21:9–15. 10.7874/jao.2017.21.1.9 28417102 PMC5392005

[17] Tripathi P Deshmukh P. Sudden sensorineural hearing loss: a review. Cureus. (2022) 14:e29458. 10.7759/cureus.29458 36299969 PMC9587755

[18] Chinese Sudden Hearing Loss Multi-Center Clinical Study Group.Prospective clinical multi-center study on the treatment of sudden deafness with different typings in China. Zhonghua Er Bi Yan Hou Tou Jing Wai Ke Za Zhi. (2013) 48:355–61. 24016557

[19] Ahmadzai N Kilty S Cheng W Esmaeilisaraji L Wolfe D Bonaparte JP. A systematic review and network meta-analysis of existing pharmacologic therapies in patients with idiopathic sudden sensorineural hearing loss. PLoS ONE. (2019) 14:e0221713. 10.1371/journal.pone.0221713 31498809 PMC6733451

[20] Li J Ding L. Effectiveness of steroid treatment for sudden sensorineural hearing loss: a meta-analysis of randomized controlled trials. Ann Pharmacother. (2020) 1060028020908067. 10.1177/1060028020908067 32126823

[21] Li Y Kanzaki S Shibata S Nakamura M Ozaki M Okano H. Comparison of inner ear drug availability of combined treatment with systemic or local drug injections alone. Neurosci Res. (2020) 155:27–33. 10.1016/j.neures.2019.07.001 31278973

[22] Mirian C Ovesen T. Intratympanic vs systemic corticosteroids in first-line treatment of idiopathic sudden sensorineural hearing loss: a systematic review and meta-analysis. JAMA Otolaryngol Head Neck Surg. (2020) 146:1–8. 10.1001/jamaoto.2020.0047 32163109 PMC7068668

[23] Demirhan H Gökduman AR Hamit B Yürekli Altindag MF Yigit Ö. Contribution of intratympanic steroids in the primary treatment of sudden hearing loss. Acta Otolaryngol. (2018) 138:648–51. 10.1080/00016489.2018.1438660 29513064

[24] Ng JH Ho RC Cheong CS Ng A Yuen HW Ngo RY. Intratympanic steroids as a salvage treatment for sudden sensorineural hearing loss? A meta-analysis. Eur Arch Otorhinolaryngol. (2015) 272:2777–82. 10.1007/s00405-014-3288-8 25217083

[25] Wang Y Han L Diao T Jing Y Wang L Zheng H. A comparison of systemic and local dexamethasone administration: from perilymph/cochlea concentration to cochlear distribution. Hear Res. (2018) 370:1–10. 10.1016/j.heares.2018.09.002 30223171

[26] Ren G Xu J Lan L Ma B Zhang Q. Postauricular injection of methylprednisolone sodium succinate as a salvage treatment for refractory sudden sensorineural hearing loss. Ir J Med Sci. (2021) 190:1165–72. 10.1007/s11845-021-02610-6 33866519

[27] Lv L Gao Z Liu J Zhuang Y Hou J Zhu W. Comparison between postauricular steroid injection and intratympanic steroid perfusion for refractory severe and profound sudden sensorineural hearing loss. Am J Otolaryngol. (2022) 43:103189. 10.1016/j.amjoto.2021.103189 34492426

